# Enhanced activity of multiple TRIC‐B channels: an endoplasmic reticulum/sarcoplasmic reticulum mechanism to boost counterion currents

**DOI:** 10.1113/JP277241

**Published:** 2019-04-14

**Authors:** Fiona O'Brien, David Eberhardt, Katja Witschas, Sam El‐Ajouz, Tsunaki Iida, Miyuki Nishi, Hiroshi Takeshima, Rebecca Sitsapesan, Elisa Venturi

**Affiliations:** ^1^ Department of Pharmacology University of Oxford Oxford UK; ^2^ Graduate School of Pharmaceutical Sciences Kyoto University Kyoto Japan

**Keywords:** Ca^2+^ release, TRIC channels, sarcoplasmic reticulum, Ryanodine receptor

## Abstract

**Key points:**

- There are two subtypes of trimeric intracellular cation (TRIC) channels but their distinct single‐channel properties and physiological regulation have not been characterized. We examined the differences in function between native skeletal muscle sarcoplasmic reticulum (SR) K^+^‐channels from wild‐type (WT) mice (where TRIC‐A is the principal subtype) and from *Tric‐a* knockout (KO) mice that only express TRIC‐B.
- We find that lone SR K^+^‐channels from *Tric‐a* KO mice have a lower open probability and gate more frequently in subconducting states than channels from WT mice but, unlike channels from WT mice, multiple channels gate with high open probability with a more than six‐fold increase in activity when four channels are present in the bilayer.
- No evidence was found for a direct gating interaction between ryanodine receptor and SR K^+^‐channels in *Tric‐a* KO SR, suggesting that TRIC‐B–TRIC‐B interactions are highly specific and may be important for meeting counterion requirements during excitation–contraction coupling in tissues where TRIC‐A is sparse or absent.

**Abstract:**

The trimeric intracellular cation channels, TRIC‐A and TRIC‐B, represent two subtypes of sarcoplasmic reticulum (SR) K^+^‐channel but their individual functional roles are unknown. We therefore compared the biophysical properties of SR K^+^‐channels derived from the skeletal muscle of wild‐type (WT) or *Tric‐a* knockout (KO) mice. Because TRIC‐A is the major TRIC‐subtype in skeletal muscle, WT SR will predominantly contain TRIC‐A channels, whereas *Tric‐a* KO SR will only contain TRIC‐B channels. When lone SR K^+^‐channels were incorporated into bilayers, the open probability (Po) of channels from *Tric‐a* KO mice was markedly lower than that of channels from WT mice; gating was characterized by shorter opening bursts and more frequent brief subconductance openings. However, unlike channels from WT mice, the Po of SR K^+^‐channels from *Tric‐a* KO mice increased as increasing channel numbers were present in the bilayer, driving the channels into long sojourns in the fully open state. When co‐incorporated into bilayers, ryanodine receptor channels did not directly affect the gating of SR K^+^‐channels, nor did the presence or absence of SR K^+^‐channels influence ryanodine receptor activity. We suggest that because of high expression levels in striated muscle, TRIC‐A produces most of the counterion flux required during excitation‐contraction coupling. TRIC‐B, in contrast, is sparsely expressed in most cells and, although lone TRIC‐B channels exhibit low Po, the high Po levels reached by multiple TRIC‐B channels may provide a compensatory mechanism to rapidly restore K^+^ gradients and charge differences across the SR of tissues containing few TRIC‐A channels.

## Key points

- There are two subtypes of trimeric intracellular cation (TRIC) channels but their distinct single‐channel properties and physiological regulation have not been characterized. We examined the differences in function between native skeletal muscle sarcoplasmic reticulum (SR) K^+^‐channels from wild‐type (WT) mice (where TRIC‐A is the principal subtype) and from *Tric‐a* knockout (KO) mice that only express TRIC‐B.
- We find that lone SR K^+^‐channels from *Tric‐a* KO mice have a lower open probability and gate more frequently in subconducting states than channels from WT mice but, unlike channels from WT mice, multiple channels gate with high open probability with a more than six‐fold increase in activity when four channels are present in the bilayer.
- No evidence was found for a direct gating interaction between ryanodine receptor and SR K^+^‐channels in *Tric‐a* KO SR, suggesting that TRIC‐B–TRIC‐B interactions are highly specific and may be important for meeting counterion requirements during excitation–contraction coupling in tissues where TRIC‐A is sparse or absent.

## Introduction

Intracellular Ca^2+^ release from the sarcoplasmic reticulum (SR) or endoplasmic reticulum (ER) depends primarily on two types of Ca^2+^ release channel, the ryanodine receptor (RyR) and the inositol trisphosphate receptor (IP_3_R). In skeletal and cardiac muscle, where rapid release of large amounts of SR Ca^2+^ are required to cause muscle contraction, RyR is the main pathway for Ca^2+^ release and the isoforms participating are primarily RyR1 in skeletal and RyR2 in cardiac muscle (Bers 2001). In other tissues, SR/ER Ca^2+^‐release may be mediated solely by IP_3_R or may involve both IP_3_R and RyR (Berridge 2016). In all cases, the positive charge that moves out of the SR/ER as Ca^2+^ is released must be compensated for by counterion movement to maintain the SR/ER membrane potential near 0 mV. Inefficient charge compensation would allow the SR/ER membrane potential to approach the Ca^2+^ reversal potential, thus terminating SR/ER Ca^2+^‐release prematurely. Following the Ca^2+^ release process, any participating counterion must be re‐equilibrated across the SR and charge compensation is again required as Ca^2+^ is pumped back into the SR/ER.

Data gathered in the 1970s and 1980s provided strong evidence for efficient counterion fluxes during SR Ca^2+^ release in skeletal muscle (Somlyo *et al*. 1977; Miller 1978; Labarca & Miller 1981; Somlyo *et al*. 1981; Garcia & Miller 1984; Somlyo *et al*. 1985). By comparing ion flux measurements from isolated SR vesicles with the conductance and permeability properties of skeletal SR K^+^‐channels incorporated into artificial membranes, Garcia & Miller (1984) concluded that the SR membrane potential did not deviate significantly from 0 mV and that K^+^ flux via the SR K^+^‐channel was the most important mechanism for charge compensation (Garcia & Miller 1984). In confirmation, Sanchez *et al*. (2018) recently targeted voltage‐sensing fluorescence resonance energy transfer biosensors to mouse skeletal muscle SR membranes and also found no evidence for a measurable SR voltage change during muscle contraction (Sanchez *et al*. 2018).

Identifying the contribution played by SR K^+^‐channels in controlling SR membrane potential became more feasible after Yazawa *et al* (2007) identified two structurally related proteins in rabbit skeletal SR termed the trimeric intracellular cation channels (TRIC‐A and TRIC‐B) (Yazawa *et al*. 2007). Both proteins, when purified, exhibited common functional characteristics with native muscle SR K^+^‐channels, including monovalent cation selectivity, subconductance state gating and sensitivity to voltage (Miller 1978; Gray & Williams 1985; Hill *et al*. 1990; Yazawa *et al*. 2007; Pitt *et al*. 2010; Venturi *et al*. 2013). The development of *Tric* knockout (KO) mice demonstrated that TRIC channels were essential for the normal structure and function of many different cell types; the double *Tric* KO mouse is embryonically lethal, the *Tric‐b* KO mouse dies after birth in respiratory failure, and the *Tric‐a* KO mouse has several reported defects including SR Ca^2+^ overload and altered Ca^2+^ homeostasis in skeletal and vascular smooth muscle cells (Yazawa *et al*. 2007; Zhao *et al*. 2010; Yamazaki *et al*. 2011). Solving the structures of two TRIC‐B isoforms from the nematode proved the trimeric nature of TRIC‐B and demonstrated that each monomer contained a fluid filled ion permeation pathway (Yang *et al*. 2016). This important feature has subsequently been reported in the structures of several types of prokaryotic (Kasuya *et al*. 2016; Su *et al*. 2017) TRIC‐B channels and, more recently, in vertebrate TRIC‐A and TRIC‐B channels (Wang *et al*. 2019).

A significant role for SR K^+^‐channels in SR charge compensation was questioned when it was discovered that RyR and IP_3_R could also contribute counterion current because of their relatively poor selectivity for divalent over monovalent cations (relative permeability pCa^2+^: pK^+^∼7) (Gillespie & Fill 2008). However, recent mathematical modelling of skeletal muscle cells now supports the early hypothesis by Garcia & Miller (1984) proposing that skeletal SR K^+^‐channel flux carries the bulk of counterion current during Ca^2+^ release (Zsolnay *et al*. 2018) and, moreover, indicates that in the *Tric‐a* KO mouse, substantial deviation of the SR potential away from 0 mV would result.

If the role of SR K^+^‐channels is to provide charge compensation for Ca^2+^ movements across the SR, the question arises as to why two isoforms of TRIC are required. One might expect that only a very primitive type of ion channel would suffice: one that is selective for monovalent cations, exhibits voltage sensitivity and can open at potentials close to 0 mV. TRIC‐A appears to be particularly important in excitable mammalian tissues such as skeletal muscle, heart and brain where it is highly expressed. (Yazawa *et al*. 2007; Pitt *et al*. 2010). TRIC‐A is not detected in all tissues, whereas TRIC‐B, although usually found at low levels, is much more widespread and mutations in this gene are associated with osteogenesis imperfecta (Volodarsky *et al*. 2013; Rubinato *et al*. 2014). We therefore set out to investigate whether the permeability properties or regulation of gating of TRIC‐A and TRIC‐B were significantly different. The reported single‐channel properties of purified TRIC proteins vary markedly between investigators (Pitt *et al*. 2010; Kasuya *et al*. 2016; Yang *et al*. 2016; Su *et al*. 2017), possibly as a result of protein damage during purification by detergents or other harsh procedures. To examine TRIC channel regulation more carefully, we characterized the gating of native SR K^+^‐channels incorporated into bilayers by fusing SR vesicles from wild‐type (WT) or *Tric‐a* KO mice, thus avoiding detergents and long isolation protocols. Calculations made from western blotting and [^3^H]ryanodine binding set a crude estimate of TRIC‐A:TRIC‐B:RyR stoichiometry in skeletal muscle as ≥5:1:1; thus, we will only observe TRIC‐B from *Tric‐a* KO SR but expect to observe mainly TRIC‐A from WT SR.

The results of the present study suggest that, although the gating and permeability properties of TRIC‐A and TRIC‐B appear to be similar, the mechanisms by which stable high open probability (Po) is achieved are very different. Although channels derived from WT and *Tric‐a* KO tissue are both voltage‐sensitive and gate into multiple subconducting levels, clusters of channels from *Tric‐a* KO display an altered gating behaviour and can reach high Po levels, indicating that TRIC‐B channels may functionally interact to boost their activity, whereas TRIC‐A channels do not.

## Methods

### Ethical approval

All experiments carried out in the present study conform with the principles and regulations described in Grundy (2015). All experiments were conducted with the approval of the Animal Research Committee according to the regulations on animal experimentation at Kyoto University (Agreement no. 11–6). Mice were housed under a 12 h:12 h light/dark cycle at 22–24 °C in a controlled facility with access to food and water available *ad libitum*. *Tric‐a* KO mice with a mixed genetic background of C57Bl6 and 129/Sv were produced as described previously (Yazawa *et al*. 2007). Mixed sex *Tric‐a* KO and WT littermates (aged 8–30 weeks) were killed via isoflurane‐induced anaesthesia, followed by cervical dislocation. *Tric‐a* KO or WT skeletal muscles were dissected, snap‐frozen in liquid nitrogen and stored at –80°C.

### Isolation of SR vesicles and single‐channel recordings

SR membrane vesicles were isolated from *Tric‐a* KO mouse skeletal muscle as described previously (Sitsapesan *et al*. 1991). After incorporation of SR vesicles into lipid bilayers, K^+^ channel current fluctuations were recorded under voltage‐clamp conditions in solutions of 210 mm KPIPES and 10 μm free Ca^2+^ at pH 7.2 (Venturi *et al*. 2013). The *trans* chamber was held at ground and the *cis* chamber was clamped at various potentials relative to ground. Evidence suggests that SR vesicles incorporate into bilayers in a consistent orientation (Sitsapesan *et al*. 1991) such that the *cis* chamber corresponds to the cytosolic face of the SR channels and the *trans* chamber corresponds to the luminal side. No observable differences in vesicle fusion rates between *Tric‐a* KO and WT tissue were detected. Experiments were performed at room temperature (22 ± 2°C). The free [Ca^2+^] and pH of the solutions were determined using a Ca^2+^ electrode (Orion 93‐20;Thermo Fisher Scientific, Waltham, MA, USA) and a Ross‐type pH electrode (Orion 81‐55; Thermo Fisher Scientific) as described previously (Sitsapesan *et al*. 1991). Single‐channel recordings were digitized at 20 kHz and recorded on a computer using WinEDR 3.05 software (John Dempster, Strathclyde University, Glasgow, UK). Po measurements at ±30 mV were started 2 s after a change in holding potential to allow for the decline of the capacitance spike. We previously observed no significant time‐dependent changes in Po over time (Matyjaszkiewicz *et al*. 2015). Single‐channel current amplitudes were measured using manually controlled cursors in WinEDR and conductance values were obtained by linear regression (Prism, version 4; Graphpad Software Inc., La Jolla, USA).

### Analysis of SR K^+^‐channel activity

We previously demonstrated, for channels from *Tric‐a* KO tissue, that at least 99.9% of all SR K^+^‐channel transitions between the full open and closed states, pass through a subconducting state (Matyjaszkiewicz *et al*. 2015). In the present study, we performed the same analysis for channels derived from WT tissue. We individually inspected all openings from five separate experiments and increased the filter cut‐off frequency up to 2.5 kHz for those events that initially appeared to transition directly between full open and closed levels without first passing through a subconducting level. We found that 99.7% (6067 of 6084) of opening or closing events did pass through a subconducting level. We therefore assume that a simplified linear three state Markov model is a good approximation of SR K^+^‐channel gating regardless of whether derived from WT or *Tric‐a* KO SR:

closed C↔sub-statesS↔ open O

We therefore used this model in our analysis. We have shown that, although SR K^+^‐channels from *Tric‐a* KO mice appear to transition between multiple distinguishable sublevels, by classifying these as a single noisy conductance state (S) midway between full open (O) and closed (C) levels, with noise level (standard deviation in QuB) equal to one‐half this amplitude, we can correctly assign 99% of events as closed, subconductance and full open states (Matyjaszkiewicz *et al*. 2015). We therefore use this analysis to distinguish full openings from subconductance openings.

Single‐channel recordings for WT and *Tric‐a* knockout tissue were analysed as described previously (Venturi *et al*. 2013; Matyjaszkiewicz *et al*. 2015). Briefly, traces were digitally filtered (Gaussian Filter with cut‐off frequency of 1 kHz) and resampled at 10 kHz prior to idealization. Using the simplified linear model described above, single channel gating events were idealized using the segmental *k*‐means algorithm (Qin 2004) in the QuB software suite (State University of New York, Buffalo, NY, USA). Po was measured from 3 min of continuous recordings and the probability of dwelling in each of the states (closed, substates and open) was determined from the segmental *k*‐means idealization. Mean open times and frequency of open events were calculated from idealizations where only a single SR K^+^‐channel was gating in the bilayer and, for this analysis, events shorter than 0.6 ms were stripped from the idealized event sequences in QuB.

Lifetime distributions were computed from QuB idealizations of single SR K^+^‐channels where events shorter than 0.6 ms were stripped. Dwell times for the closed and full open state were log binned according to the methods described in Sigworth and Sine (1987) and fitted with an exponential log probability density function in Clampfit, version 10.3 (Molecular Devices, Sunnydale CA, USA). The optimal number of time constants for each distribution was determined using a log‐likelihood ratio test in Clampfit, version 10.3.

### Analysis of recordings containing more than one ion‐channel species

In experiments when both an RyR and an SR K^+^‐channel co‐incorporated into the bilayer, separation of RyR‐events from SR K^+^‐channel‐events was required to determine the Po of each channel species. As shown in Fig. 6 *B*, the current amplitude of the SR K^+^‐channel full open state is ∼25% of that of the RyR open state. Therefore, in experiments where an RyR and one SR K^+^‐channel were gating, we could still use the established 50% threshold method (Colquhoun & Sigworth 1983) in pClamp (Molecular Devices) to calculate RyR Po.

To correctly analyse SR K^+^‐channel activity from these multispecies recordings, we built a novel model in QuB. Filtering at 4 kHz (which is necessary to resolve RyR events) resulted in the subconductance states of the SR K^+^‐channel being poorly resolved and so the ‘merged’ subconductance level of the SR K^+^‐channel was not included in the model. We therefore constructed a four state Markov model consisting of the zero current level (level 1), the full open level of the SR K^+^‐channel (level 2), the RyR open level (level 3) and the level that would be observed if both isoforms were open simultaneously (level 4) (Fig. 6 *A*). The resulting idealization of each event were checked manually to ensure that single‐channel events were classified correctly. In experiments where two SR K^+^‐channels were gating in addition to an RyR, we merely added one additional state to the model (the level that would occur if two SR K^+^‐channels gated together). To determine the number of SR K^+^‐channels present in the bilayer, high positive holding potentials (≥ +60 mV) were applied for short periods of time to maximally activate the channels at the end of the experiment.

### Statistical analysis

Data are reported as the mean ± SD, where *n* = 3, or the mean ± SEM, where *n* ≥ 4. Differences between mean values were assessed using a Student's *t* test. Where multiple treatments were compared, ANOVA followed by a modified *t* test was used. *P *< 0.05 was considered statistically significant.

### Materials

All chemicals were purchased from VWR (Poole, UK) or Sigma‐Aldrich (Gillingham, UK). All solutions were prepared in MilliQ deionized water (Millipore, Harrow, UK) and those for use in bilayer experiments were filtered through a membrane with 0.45 μm pore diameter (Millipore).

## Results

**Figure 1:**
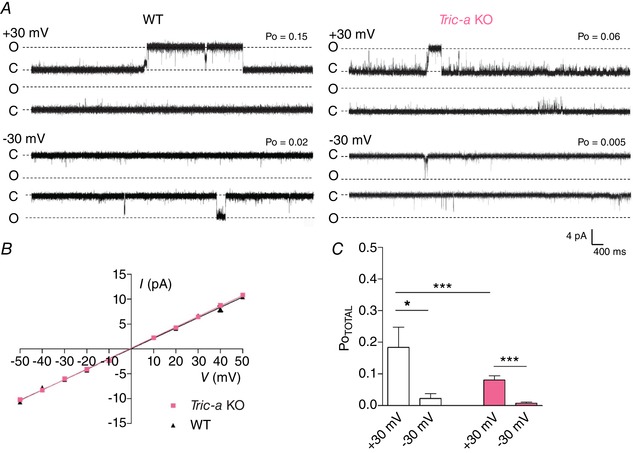
Single‐channel properties of SR K^+^‐channels from WT and *Tric‐a* KO mouse skeletal muscle *A*, representative single‐channel current fluctuations through mouse skeletal SR K^+^‐channels from WT (left) and *Tric‐a* KO (right) mice at holding potentials of ±30 mV. The recordings are representative of 12 WT and 22 *Tric‐a* KO experiments. The fully open and closed channel levels are indicated by O and C. *B*, *I*–*V* relationships for SR K^+^‐channels from WT (black triangles) and *Tric‐a* KO (pink squares) mouse skeletal muscle. *C*, overall Po (Po_TOTAL_) of SR K^+^‐channels from WT (white) and *Tric‐a* KO (pink) mouse skeletal muscle at holding potentials of ±30 mV are compared. The mean ± SEM is shown (*n* = 12 for WT and 22 for *Tric‐a* KO); where not shown, error bars are within the symbols. [Color figure can be viewed at wileyonlinelibrary.com]

The typical features of SR K^+^‐channel gating are shown in Fig. 1 *A* for channels derived from WT or *Tric‐a* KO mice. The single channel conductances of the full open state in 210 mm KPIPES (WT = 210.8 ± 2.7 pS, *n* = 25 and *Tric‐a* KO = 211.1 ± 1.8 pS, *n* = 33) were similar (Fig. 1 *B*), as were the general characteristics of gating. Fig. 1 *A* compares the gating behaviour of the two groups of channels when bilayers contained only one channel in the bilayer. Both groups of channels gated to brief subconductance open states and to longer bursts of openings to the full open state. Both groups of channels exhibited voltage‐dependent gating, being significantly less open at  −30 mV than +30 mV (Fig. 1 *C*).

**Figure 2:**
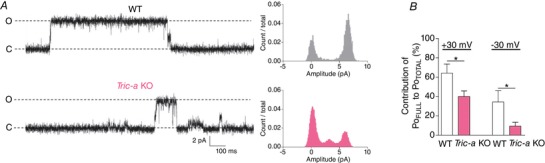
Gating characteristics of SR K^+^‐channels from WT and *Tric‐a* KO mouse skeletal muscle *A*, typical opening bursts shown on an extended time base at +30 mV and corresponding amplitude histograms for channels from WT (top) and *Tric‐a* KO (bottom) traces. The fully open channel level is indicated by O and the closed level by C. *B*, percentage contribution of Po in the full open state to the Po_TOTAL_ at +30 mV and –30 mV. The mean ± SEM is shown (*n* = 12 for WT and 22 for *Tric‐a* KO). [Color figure can be viewed at wileyonlinelibrary.com]

Although the general features of gating were similar, the channels from *Tric‐a* KO mice displayed significantly lower Po compared to those from WT mice (Fig. 1 *C*). Expanding the time base of the opening bursts (Fig. 2 *A*) allows a more detailed visualization of the complex gating of SR K^+^‐channels. The duration of full open events appeared shorter in channels derived from the skeletal muscle of *Tric‐a* KO mice. We investigated this in more detail and found that, at both positive and negative holding potentials, the openings to the full open state contributed significantly less to the overall Po of channels from *Tric‐a* KO mice compared to that observed for channels from WT mice (Fig. 2 *B*). This can also be visualized from the all‐point amplitude histograms for channels from WT and *Tric‐a* KO SR (Fig. 2 *A*).

**Figure 3:**
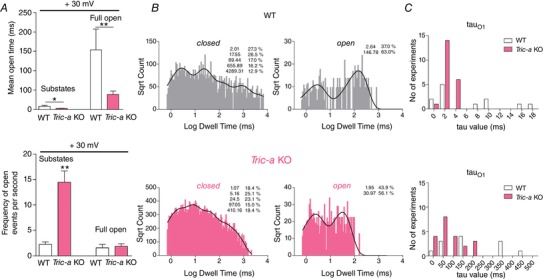
Lifetime analysis of the SR K^+^‐channels from WT and *Tric‐a* KO mouse skeletal muscle *A*, mean open time for the subconductance open states and the full open state at +30 mV (top) and frequency of openings to subconductance states and to the full open state at +30 mV (bottom). *B*, open and closed lifetime distributions and probability density functions for a representative SR K^+^‐channel from WT and from *Tric‐a* KO. The corresponding time constants (*taus*) and percentage areas are shown. *C*, frequency histogram distributions of the two open lifetime constants: tau_O1_ and tau_O2_. WT data are shown in white and *Tric‐a* KO in pink. The mean ± SEM (^*^ *P *< 0.05, ^**^ *P *< 0.01) is shown where *n* = 12 (WT) or 21 (*Tric‐a* KO). [Color figure can be viewed at wileyonlinelibrary.com]

The mean open time (MOT) of both the full open and ‘merged’ subconductance states was significantly lower in channels from *Tric‐a* KO mice (Fig. 3 *A*). In addition, we observed a marked increase in the frequency of subconducting states in SR K^+^‐channels from *Tric‐a* KO (Fig. 3 *A*). The frequency of the full open events was not significantly altered. A more detailed analysis of the full open and closed lifetime distributions (Fig. 3 *B*) was then performed. Lifetime analysis was impractical at –30 mV because there were too few events but, at +30 mV, the optimum number of time constants (*taus*) was 5 and 2 for the closed and full open channel levels, respectively (observed in 7 of 12 experiments from WT and in 15 of 21 experiments from *Tric‐a* KO). The remaining channels were refit to meet these restraints and one experiment from *Tric‐a* KO was not included because there were too few events for this depth of analysis. Compared to WT, the SR K^+^‐channel from *Tric‐a* KO had a marked reduction of both open time constants and a significant decrease of the shortest closed lifetime constant (Table 1). The MOT of the full open states [150 ± 54 ms for WT (SEM, *n* = 12) *vs*. 39 ± 9 for *Tric‐a* KO (SEM, *n* = 22)] were much longer than the MOT of subconductance states [7.9 ± 2.6 ms for WT (SEM, *n* = 12) *vs*. 3 ± 0.29 for *Tric‐a* KO (SEM, *n* = 22)]; therefore, it is this reduction in both full open lifetime constants seen in channels from *Tric‐a* KO mice that plays the largest role in determining Po. If there are approximately five TRIC‐A trimers to one TRIC‐B trimer in WT SR (Pitt *et al*. 2010; Zhao *et al*. 2010), one might expect to observe only one population of open lifetime constants (tau_O1_ and tau_O2_) from *Tric‐a* KO SR but two populations from WT SR, with the shortest being of similar duration to that of *Tric‐a* KO SR. We therefore constructed two frequency histograms showing the distributions of the two open lifetime constants, tau_O1_ and tau_O2_ (Fig. 3 *C*), for both WT and *Tric‐a* KO groups. The histograms show that SR K^+^‐channels from WT SR exhibited a wider range of open lifetime constants, indicating that there could be more than one population of channels within this group.

**Table 1:** Time constants and percentage areas are shown as obtained from maximum likelihood fitting of probability density functions to full open and closed lifetime distributions of single SR K^+^‐channels from WT and *Tric‐a* KO

WT		*Tric‐a* KO	
tau (ms)	Area (%)	tau (ms)	Area (%)
Closed state			
5.2 ± 2.2	19.4 ± 3.1	1.3 ± 0.1*	23.2 ± 1.7
25.6 ± 17.7	21.3 ± 2.7	10.6 ± 2.5	21.2 ± 1.9
138.6 ± 72.6	27.5 ± 4.7	65.6 ± 17.6	20.3 ± 1.5
810.2 ± 319.7	20.2 ± 3.4	766.9 ± 406.9	17.8 ± 2.4
4403.9 ± 1666.1	11.6 ± 2.0	6601.0 ± 2841.2	17.5 ± 2.2
Full open state			
6.4 ± 2.0	54.3 ± 9.2	2.4 ± 0.2*	54.2 ± 3.7
182.3 ± 48.7	45.6 ± 9.2	81.7 ± 14.3*	45.8 ± 3.7

Data are presented as the mean ± SEM for *n* = 12 WT experiments and *n* = 21 *Tric*‐*a* KO experiments. Student's *t* test was used to assess differences between mean tau values of the two groups (^*^ *P *< 0.05).

**Figure 4:**
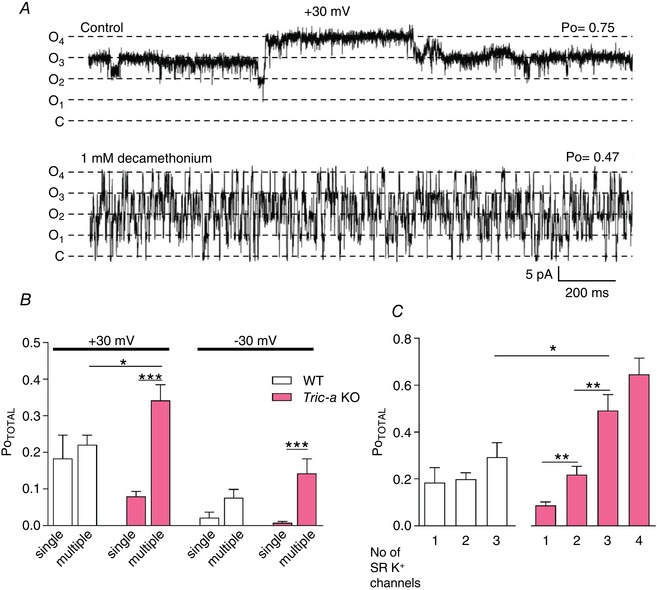
Comparison of the activity of single *vs*. multiple SR K^+^‐channels *A*, decamethonium was used to count channels in the bilayer. The top trace shows the control current fluctuations through multiple SR K^+^‐channels. After addition of 1 mm cytosolic decamethonium, rapid blocking events are observed without lowering the current amplitude and it is clear that the bilayer contains four SR K^+^‐channels with no observable leak currents. *B*, overall Po (Po_TOTAL_) of channels, at ±30 mV, derived from WT or *Tric‐a* KO mice when multiple channels were present in the bilayer in comparison with the Po when only a single channel was present. *C*, data are further divided into experiments where only one, two, three or four channels were present in the bilayer and the average Po_TOTAL_ is shown for WT and *Tric‐a* KO experiments at +30 mV. For WT, there were 12 experiments with one channel, 31 experiments with two channels and 7 experiments with three channels in the bilayer. For *Tric‐a* KO, there were 22 experiments with one channel, 17 experiments with two channels, 6 experiments with three channels and 4 experiments with four channels in the bilayer. ^*^ *P *< 0.05, ^**^ *P *< 0.01, ^***^ *P *< 0.01. [Color figure can be viewed at wileyonlinelibrary.com]

Using the simplified noisy substate linear gating model (C ↔ S ↔ O), we are not restricted to analysing experiments where a single SR vesicle incorporation resulted in only one actively gating SR K^+^‐channel. We therefore also analysed those experiments where up to four SR K^+^‐channels were gating after a single vesicle fusion. It was very difficult to obtain recordings where there were clearly only four SR K^+^‐channels in the bilayer and we did not obtain reliable recordings from WT tissue with this number of channels. Fig. 4 *A* (top trace) is typical of the high Po that was observed when four channels were present in the bilayer after incorporation of SR from *Tric‐a* KO mice. To accurately count the numbers of channels in the bilayer, we added decamethonium (1 mm), a known SR K^+^‐channel blocker (Coronado & Miller 1980; Miller 1982), to the cytosolic channel side at the end of the experiment, as shown in Fig. 4 *A* (bottom trace). This concentration of decamethonium does not affect single‐channel conductance but causes frequent blocking events and reduces Po (*n* = 3, *P *< 0.01, data not shown). This enables us to determine where the zero current level is and hence the number of actively gating channels. We thus remove any possibility that a non‐selective leak in the bilayer could lead to an overestimation of the total number of channels.

**Figure 5:**
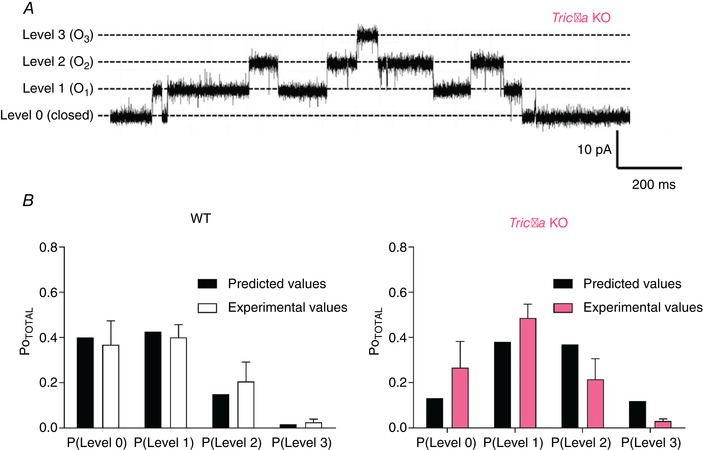
Binomial analysis for three SR K^+^‐channels in the bilayer at +30 mV *A*, a typical recording is shown where three SR K^+^‐channels were present in the bilayer showing the zero current level (all channels are simultaneously closed (level 0) and the levels where one (O1), two (O2) and three (O3) channels are simultaneously open. *B*, for experiments where three channels were present in the bilayer, the binomial statistical prediction of the probability of observing current fluctuations to each level is shown (black bars). Predicted values were calculated using the Po derived from the respective experiments where three SR K^+^‐channels were present in the bilayer (WT Po = 0.29, *Tric‐a* KO Po = 0.49) (Fig. 4 *C*). The white (WT) and pink (*Tric‐a* KO) bars represent the actual experimental data. The mean ± SEM is shown for WT (*n* = 7) and *Tric‐a* KO (*n* = 6). [Color figure can be viewed at wileyonlinelibrary.com]

Figure 4 *B* shows that, although the average Po of multiple channels from WT mice was similar to that for the single channels, this was not so for channels from *Tric‐a* KO mice where the Po of multiple channels was significantly higher than that of single channels. The Po of multiple channels from *Tric‐a* KO mice was even higher than the Po of multiple channels from WT mice. We therefore split the data further into experiments where one, two, three or four channels were present in the bilayer (Fig. 4 *C*). The Po of each channel derived from WT mice appeared to be independent of the number of channels incorporated into the bilayer. By contrast, the Po of each channel from *Tric‐a* KO mice increased as the number of channels in the bilayer increased suggesting that the presence of one channel facilitates the opening of other channels in the bilayer. In addition, subconductance gating appeared altered in multichannel recordings. Although, in lone channels from *Tric‐a* KO SR, the majority of openings were to subconducting levels (∼60 %) (Fig. 2 *B*), these decreased to only ∼22% when three SR K^+^ ‐channels were present (*n* = 22 for singles and *n* = 6 for three‐channel recordings, *P *< 0.01, data not shown). Note that, in those recordings where three SR K^+^‐channels were actively gating in the bilayer, the average Po of each channel from *Tric‐a* KO mice was significantly greater than the average Po from WT channels (WT Po = 0.29, *n* = 7 and *Tric‐a* KO Po = 0.49, *n* = 6, *P *< 0.05). In these three‐channel recordings, four possible current levels may be observed. These include the levels where all three channels are closed (level 0) and where one (level O_1_), two (level O_2_) and three (level O_3_) channels are open (Fig. 5 *A*). To test whether multiple channels from *Tric‐a* KO or WT SR gate independently or in a co‐operative manner, we calculated the binomial distribution of the occupancies for the closed and open levels using the average Po for recordings with three actively gating channels. Fig. 5 *B* shows that the mean experimental occupancies follow the binomial predictions for both groups of channels suggesting that multiple TRIC‐B channels gate at an intrinsically higher Po level but do not appear to gate co‐operatively.

**Figure 6:**
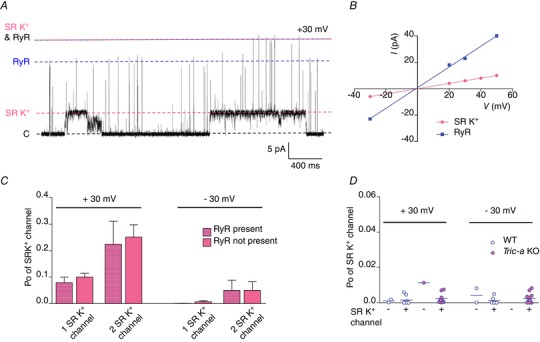
SR K^+^‐channels gating in the presence of RyR *A*, an example of a recording where an RyR and an SR K^+^‐channel are gating simultaneously in the bilayer. The dashed lines indicate the current levels when all channels are closed (black), or where one SR K^+^‐channel only (pink), one RyR channel only (blue) or both an RyR and an SR K^+^‐channel (blue and pink) are open. *B*, *I*–*V* relationships for RyR and SR K^+^‐channels. *C*, comparison of SR K^+^‐channel Po in the presence and absence of a RyR channel. *D*, comparison of RyR channel gating when activated only by 10 μm cytosolic Ca^2+^, for both WT and *Tric‐a* KO tissue at ±30 mV and in the presence and absence of SR K^+^‐channels. [Color figure can be viewed at wileyonlinelibrary.com]

Because multiple TRIC‐B channels, when actively gating in the bilayer display an altered gating profile, we investigated the possibility that other species of ion‐channels that co‐incorporated into the bilayer during SR vesicle fusion could also influence TRIC‐B gating. In KPIPES solutions, we can observe both RyR and SR K^+^‐channels gating simultaneously in the same bilayer (Fig. 6 *A*). We therefore examined whether an RyR gating in the bilayer altered the gating of SR K^+^‐channels from *Tric‐a* KO mice. The single‐channel conductance of RyR1 is so much greater than that of an SR K^+^‐channel (Fig. 6 *B*) that the openings of the SR K^+^‐channel can still be determined using QuB software. We used bilayers where there was one RyR and either one or two SR K^+^‐channels; however, the presence of RyR did not appear to alter SR K^+^‐channel activity at either ±30 mV (Fig. 6 *C*). We next examined if the SR K^+^‐channels could influence RyR activity. Fig. 6 *D* compares the Po of RyR channels from WT or *Tric‐a* KO mice where there was either no SR K^+^‐channel or at least one SR K^+^‐channel in the bilayer. We performed the analysis at ±30 mV. While we frequently observed bilayer fusion events that incorporated SR K^+^‐channels without RyR, we only observed RyR incorporation without SR K^+^‐channels in three bilayers in total, indicating that there are many more SR K^+^‐channels than RyR in the junctional SR membranes. Under these experimental conditions, with 10 μm cytosolic Ca^2+^ as the only activating ligand and with SR K^+^‐channels gating simultaneously in the bilayer, the Po of RyR from WT or *Tric‐a* KO mice was similar at both +30 mV and –30 mV. Because SR K^+^‐channels are voltage‐dependent and gate with very low Po at –30 mV (Fig. 6 *C*), this suggests that the presence of SR K^+^‐channels does not directly affect the opening of RyR channels. In those three bilayers (2 × WT and 1 × *Tric‐a* KO) where there were no SR K^+^‐channels present, RyR Po appeared similar to that observed where there were SR K^+^‐channels present.

## Discussion

We demonstrate that the SR K^+^‐channels from *Tric‐a* KO mice behave differently from those derived from WT mice. Although the single channel conductance is similar, and the channels retain sensitivity to voltage, the Po of the channels from *Tric‐a* KO mice is lower if those channels are single channels gating alone in the bilayer. This is primarily because the channels gate with shorter full open dwell times so that the subconductance openings contribute more to the overall Po. When we widened our analysis to include bilayers where multiple channels had incorporated, we found that the channels derived from *Tric‐a* KO mice exhibited a markedly higher Po as the number of channels in the bilayer was increased, a feature not observed with channels from WT SR.

We should consider what enhanced multiple channel activity might mean in the light of the recent independently reported crystal structures of TRIC (Kasuya *et al*. 2016; Yang *et al*. 2016; Su *et al*. 2017; Wang *et al*. 2019) and in the light of what SR K^+^‐channel ‘subconductance state’ implies. The purified proteins used to obtain crystals of TRIC‐B (TRIC‐B1 and TRIC‐B2 from *Caenorhabditis elegans* (CeTRIC‐B1 and CeTRIC‐B2) (Yang *et al*. 2016); prokaryotic SaTRIC‐B from *Sulfolobusacidocaldarius* and CpTRIC‐B from *Colwelliapsychrerythraea* (Su *et al*. 2017); prokaryotic RsTRIC‐B from *Rhodobactersphaeroides* and SsTRIC‐B from *Sulfolobussolfataricus* (Kasuya *et al*. 2016); chicken TRIC‐A (GgTRIC‐A) and frog TRIC‐B (XlTRIC‐B) (Wang *et al*. 2019) were also investigated for their single‐channel properties. The conductance and gating properties from these studies vary extensively but, fortunately, three of the studies (Yang *et al*. 2016; Su *et al*. 2017; Wang *et al*. 2019) used exactly the same K^+^ concentration (symmetrical 210 mm K^+^) as that used in our previous and current reports of native (Venturi *et al*. 2013; Matyjaszkiewicz *et al*. 2015) or purified (Pitt *et al*. 2010) TRIC channel function. The single‐channel conductance of the full open state of CeTRIC‐B1 was reported as 153 pS with substates of 101 and 45 pS (Yang *et al*. 2016). The recordings were filtered at 50 Hz and so only long events (>20 ms) were resolved. As the substates were approximately one‐third and two‐thirds of the amplitude of the apparent full open state, it was suggested that the substates represented the opening of one or two of the pores within the trimeric channel, whereas the full open state resulted from the simultaneous opening of all three pores. The full open state of SaTRIC‐B (Su *et al*. 2017) was of similar amplitude to that of CeTRIC‐B1 in 210 mm K^+^ (153 pS), although filtering the data at 1 kHz enabled shorter events to be observed and four (not three) sublevels were reported. The same group also recently published the high resolution crystal structures for vertebrate GgTRIC‐A and XlTRIC‐B showing very close similarities to the trimeric three‐pore structures of prokaryotic TRIC (Wang *et al*. 2019). In a recent study, they reported lower and almost indistinguishable single‐channel conductances for GgTRIC‐A (128 ± 6 pS) and XlTRIC‐B (125 ± 13 pS) but subconductance gating was not discussed (Wang *et al*. 2019). RsTRIC‐B (Kasuya *et al*. 2016) was recorded in ionic conditions (symmetrical 150 mm K^+^ and 20 mm Ca^2+^) where the single‐channel conductance of TRIC‐B would be expected to be lower than in 210 mm K^+^, yet we estimate from the *I*–*V* plot shown that the apparent full open state is ∼240 pS; perhaps two channels were gating in a coupled manner.

We suggest that the variability in the above reported single‐channel properties results from the difficulty in preserving TRIC ion channel function following purification of recombinantly expressed proteins. When investigating purified, recombinant mouse TRIC‐A and TRIC‐B, we observed an apparent full open single‐channel conductance of ∼140 pS for TRIC‐B and 192 pS for TRIC‐A with wide variability around the mean values (Pitt *et al*. 2010). The 140 pS full TRIC‐B level was indistinguishable from the most frequently observed substate level of purified TRIC‐A and was similar to sublevels of native SR K^+^‐channels from the *Tric‐a* KO mouse where only TRIC‐B could be gating (Venturi *et al*. 2013). We therefore concluded that our purified mouse TRIC‐B channels were affected by the heterologous cell overexpression/purification protocols and only gated in the sublevels (Pitt *et al*. 2010). We suggest that this may also be the case for the channels purified by other groups, especially given that the conductance of the apparent full open state of CeTRIC‐B1 (Yang *et al*. 2016) and SaTRIC‐B (Su *et al*. 2017) is so similar to that of our purified mouse TRIC‐B (Pitt *et al*. 2010) and that of sublevels of native mouse SR K^+^‐channels (Venturi *et al*. 2013).

**Figure 7:**
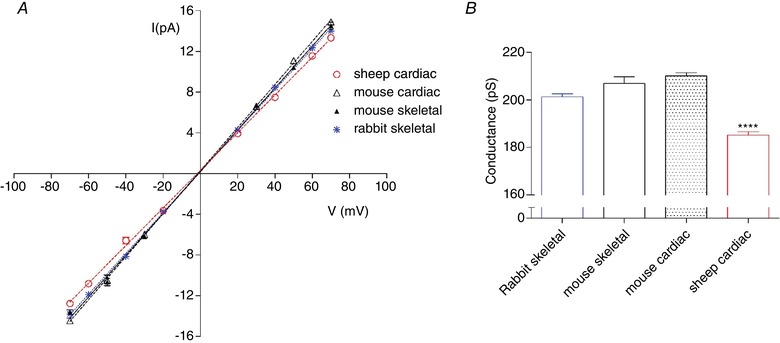
Comparison of single‐channel conductances for SR K^+^‐channels from mouse, rabbit and sheep *A*, *I*–*V* relationships for SR K^+^‐channels recorded from rabbit skeletal (blue), sheep cardiac (red open circle), mouse cardiac (black open triangle) and mouse skeletal (black filled triangle) WT muscle. *B*, mean single channel conductance. Data are the mean ± SEM (*n* = 13 for rabbit skeletal; *n* = 28 for sheep cardiac; *n* = 7 for mouse cardiac; *n* = 25 for mouse skeletal. ^****^ *P *< 0.0001). [Color figure can be viewed at wileyonlinelibrary.com]

Species differences could also explain the variability in single‐channel conductance values reported, especially given that SR K^+^‐channel conductance is known to be sensitive to temperature, pH and divalent cation concentration (Coronado *et al*. 1980; Labarca *et al*. 1980; Coronado & Miller 1982; Bell 1985; Liu & Strauss 1991; Shen *et al*. 1993). We find that, even when SR membrane vesicles are prepared using identical procedures and when recordings are made under identical experimental conditions, there are small but significant differences in the single‐channel conductances of SR K^+^‐channels from different mammalian species. Fig. 7 shows this clearly for rabbit, mouse and sheep.

**Figure 8:**
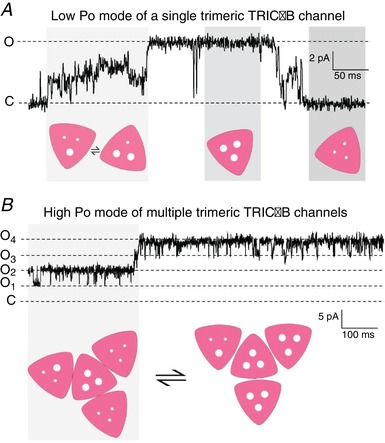
Models to explain single low Po gating *vs*. multiple high Po gating modes of TRIC‐B *A*, model illustrating how the simultaneous opening of none, one, two and three pores within a single trimeric TRIC‐B channel could give rise to the known gating characteristics of a single SR K^+^‐channel. *B*, the model suggests how multiple TRIC‐B channels, by physically interacting, could switch to a high Po mode, resulting in an increase of simultaneous openings of multiple pores within each trimeric TRIC‐B channel. [Color figure can be viewed at wileyonlinelibrary.com]

Taking into account all of the data obtained by us and other groups, how can we explain the opening of the three individual pores that make up one trimeric TRIC channel? We speculate that each mouse TRIC‐A and TRIC‐B trimer have similar full conductance amplitudes of ∼210 pS (Fig. 1) but that TRIC‐B, when lone channels are gating, prefer to open to subconductance levels rather than the full open state (Fig. 2). In this scenario, we suggest that the full open state current amplitude is only reached when three pores are open simultaneously (Fig. 8). When fewer than three pores are open, the instability of these states leads to rapid, incompletely resolved transitions out of those states giving rise to the ‘messy’ subconductance state gating that characterizes these channels (Fig. 8). The channels can even gate between substates for prolonged periods of time as reported previously by Venturi *et al*. (2013). If 210 pS represents the approximate TRIC‐B full open state (mouse), then we must assume that the enhanced channel gating that we observe in multichannel recordings (Fig. 4) is between trimeric channels rather than between three individual monomers of a single channel. We suggest that physical interactions between multiple trimeric channels change the gating behaviour of the lone channels, as illustrated in Fig. 8, where the simultaneous opening of three pores is energetically unfavourable, to a situation where the simultaneous opening of three pores is very probable, as shown in Fig. 8 for four trimeric channels gating in a high Po mode.

**Figure 9:**
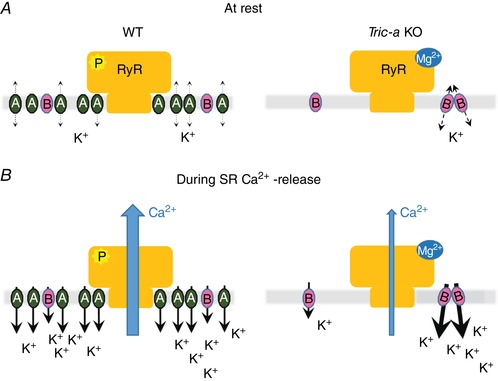
Model of the role of TRIC‐A and TRIC‐B in WT and *Tric‐a* KO skeletal muscle An approximate ratio of five TRIC‐A to one TRIC‐B: one RyR1 is assumed (Pitt *et al*. 2010; Zhao *et al*. 2010). *A*, at rest: in WT SR, when the membrane potential across SR is 0 mV, the Po of TRIC channels, particularly TRIC‐B, is low but sufficient to equilibrate K^+^ across the SR. In *Tric‐a* KO SR, where TRIC‐B channels can couple together, they pass more current than isolated channels. *B*, during SR Ca^2+^ release: in WT SR, TRIC channels open more if the SR becomes more negative. In *Tric‐a* KO, there are fewer SR K^+^‐channels that may not pass sufficient K^+^ current even though clustered TRIC‐B channels may also gate with high Po *in vivo*. RyR1 Po is already lower in *Tric‐a* KO (El‐Ajouz *et al*. 2017) as a result of increased inhibition by Mg^2+^ and the inability of phosphorylation to stimulate RyR1 opening. If there is insufficient counterion current, RyR Po will be limited even more as the reversal potential of Ca^2+^ is reached. [Color figure can be viewed at wileyonlinelibrary.com]

To understand why altered behaviour between multiple SR K^+^‐channels should emerge only when TRIC‐A is removed, we must consider the possible arrangement and expression levels of TRIC proteins in skeletal muscle heavy SR (Fig. 9). It is estimated that, for every RyR tetramer, there are approximately five TRIC‐A trimers and one TRIC‐B trimer (Pitt *et al*. 2010; Zhao *et al*. 2010) and so we would normally expect to observe TRIC‐A channels when incorporating WT SR into bilayers. Channel gating is unaffected when multiple channels are co‐incorporated and therefore we assume that no TRIC‐A‐TRIC‐A interactions that would alter channel gating are present. Likewise, when SR vesicles from *Tric‐a* KO tissue are incorporated into bilayers, the only possible TRIC‐protein that can be gating in a bilayer is TRIC‐B. When isolated single channels are present, they gate with low Po but when there are multiple channels in the bilayer, the gating is altered and channels can reach high Po levels indicating that TRIC‐B channels may be able to interact with each other. Although we do not know the nature of these interactions, the removal of TRIC‐A, a highly expressed protein may allow more opportunity for TRIC‐B─TRIC‐B interactions that were otherwise rare within a crowded SR membrane. It is possible that TRIC‐B─TRIC‐B or TRIC‐B─TRIC‐A interactions may be present also in WT tissue, although we do not observe this detail because of the overwhelming presence (more than five‐fold) of TRIC‐A.

There is no biochemical evidence that TRIC channels bind directly to each other and so any inter‐channel interactions are probably reversible and dynamic, created by opportune contact and controlled by the immediate biochemical and biophysical environment of the bilayer/SR membrane. The high Po gating, for example, could be the result of channel‐channel interactions through exposed domains or could be driven by mechanical cues transmitted by membrane perturbations arising when a nearby channel opens and closes. Movement transmitted via the membrane appears to be a less probable explanation because, when RyR channels were also present and gating in the bilayer (and presumably mechanically deforming the membrane locally), they did not appear to affect SR K^+^‐channel activity (Fig. 6), indicating that there is no functional coupling between RyR and SR K^+^‐channels. In confirmation of this, although we have few experiments where an RyR channel incorporated into the bilayer without the co‐incorporation of one or more SR K^+^‐channels, the Po of RyR does not appear to be altered by the presence of SR K^+^‐channels in the bilayer, nor by whether the SR K^+^‐channels are opening extensively, as at +30 mV, or are opening rarely, as at –30 mV.

High throughput, rapid SR Ca^2+^ release through RyR1 during EC‐coupling would not take place without counterion current to balance the loss of positive charge from the SR. Redistribution of charge is also required as Ca^2+^ is pumped back into the SR during relaxation. By mathematically modelling the ionic fluxes through the putative contributors of counterion current (Zsolnay *et al*. 2018), it has been suggested that no single species of ion channel is essential for physiological counterion current during the process of EC‐coupling. In one sense, we agree with this; in the *Tric‐a* KO mouse, even though there is normally five‐fold more TRIC‐A than TRIC‐B in skeletal SR, and although TRIC‐B is not upregulated, the mouse still survives until adulthood, albeit with defects in skeletal muscle structure and function (Zhao *et al*. 2010). However, TRIC‐A and TRIC‐B are subtypes of the same family of protein and, if both subtypes are deleted, the mouse does not survive; the *Tric* double KO mouse dies in embryonic heart failure, indicating that at least some TRIC protein is required for muscle function (Yazawa *et al*. 2007).

The results obtained in the present study indicate how the distinct and diverse gating profile of TRIC‐B may help to overcome the drastic reduction in the overall TRIC channel population that occurs in the *Tric‐a* KO mouse and this is summarized in Fig. 9. In normal skeletal muscle at rest (WT mouse), it has long been argued that the membrane potential across the SR is close to 0 mV and that there is equilibration of mobile ions (not Ca^2+^) across the SR (Somlyo *et al*. 1977; Miller 1978; Somlyo *et al*. 1981; Garcia & Miller 1984; Somlyo *et al*. 1985). At 0 mV, SR K^+^‐channel opening is probably low and predominantly to subconducting open states (Matyjaszkiewicz *et al*. 2015) but, because of the high expression levels of TRIC channels, it is expected that there would be significant monovalent cation current when the RyR were closed. In *Tric‐a* KO mice, there would be reduced potential for fast equilibration of monovalent cations because of the large reduction in overall numbers of SR K^+^‐channels. During muscle contraction in normal (WT) skeletal muscle, for every RyR1 that is releasing the SR Ca^2+^, there is the capacity for at least five or more TRIC channels to open. Any tendency for negative charge build up in the SR will increase the Po of all SR K^+^‐channels because of their voltage‐dependence; the greater the voltage change, the greater the Po of the SR K^+^‐channels. As the double *Tric* KO mouse model is lethal (Yazawa *et al*. 2007), and because skeletal muscle SR Ca^2+^ release is distorted in the *Tric‐a* KO mouse (Zhao *et al*. 2010), this suggests that RyR1 plus other contributors to counterion fluxes (such as SR Cl^‐^ channels), in the absence of TRIC channels, cannot pass sufficient counterion current during SR Ca^2+^ release to maintain normal function, hence highlighting the essential role of TRIC channels. Zsolnay *et al* (2018) also came to this conclusion with their recent mathematical modelling of SR counterion fluxes, proposing that, in the absence of SR K^+^‐channels, a build‐up of negative charge within the SR lumen would result (Zsolnay *et al*. 2018). Sanchez *et al*. (2018) have recently expressed SR targeted voltage‐sensitive fluorescence resonance energy transfer probes in skeletal muscle fibres aiming to monitor voltage changes across the SR during SR Ca^2+^ release and found that, in agreement with previous predictions, no measurable change in SR voltage occurred (Sanchez *et al*. 2018). Thus, the SR K^+^‐channel appears to be a crucial player in the process of EC‐coupling, enabling rapid and graded counterion currents to maintain SR membrane potential and support SR Ca^2+^ release through RyR channels. TRIC‐B alone does not provide sufficient K^+^ flux during skeletal muscle EC‐coupling hence the Ca^2+^ overload and irregularities of SR Ca^2+^ release in skeletal muscle derived from *Tric‐a* KO mice (Zhao *et al*. 2010). Our model (Fig. 9) shows that, even though TRIC‐B channels may physically interact to boost Po, the large overall reduction in SR K^+^‐channel numbers outweighs this benefit.

The results obtained in the present study are important for understanding the role of TRIC‐B in other tissues. TRIC‐A is very heavily expressed in cardiac and skeletal muscle and in the brain compared to TRIC‐B (Pitt *et al*. 2010; Zhao *et al*. 2010) and, therefore, in the adult, TRIC‐B may provide only a minor contribution to SR K^+^ fluxes in these tissues. TRIC‐B is present in most other cell types and, because TRIC‐A may be absent or present only at low levels in these cells, the normal functioning of TRIC‐B may be critical for physiological SR Ca^2+^ homeostasis in a range of tissues. This is manifested in the *Tric‐b* KO mouse, which dies as soon as it is born because intracellular Ca^2+^ release in the alveolar type II epithelial cells is impaired (Yamazaki *et al*. 2009). This is also borne out in the disease, osteogenesis imperfecta, where mutations to TRIC‐B cause bone fragility (Volodarsky *et al*. 2013; Rubinato *et al*. 2014). The high Po mode that clustered TRIC‐B channels display may be a key feature that allows TRIC‐B channels to open rapidly and appropriately in non‐excitable tissues, where it is the main cation channel carrying counterion currents to compensate for Ca^2+^ movements across the SR.

## Additional information

### Competing interests

The authors declare that they have no competing interests.

### Author contributions

RS and EV conceived and designed the experiments. TI, MN and HT produced and characterized *Tric‐a* KO mice and provided tissue. FO'B, DE and EV performed the SR K^+^‐channel experiments and produced the artwork. FO'B and DE analysed the SR K^+^‐channel experiments. EV, SE, FO'B and DE performed and analysed the RyR channel experiments. FO'B, EV and KW isolated SR membrane vesicles. RS wrote the article. RS and EV revised the article. All authors discussed the results and commented on the article. All of the authors approved the final version of the manuscript submitted for publication. All authors agree to be accountable for all aspects of the work in ensuring that questions related to the accuracy or integrity of any part of the work are appropriately investigated and resolved. All persons designated as authors qualify for authorship, and all those who qualify for authorship are listed.

### Funding

This work was supported by the British Heart Foundation (RG/10/114128576, FS/11/3128790, FS/13/5730647) and Japan Society for the Promotion of Science (Core‐to‐core program).
